# Integrating Two-Dimensional Gas and Liquid Chromatography-Mass Spectrometry for Untargeted Colorectal Cancer Metabolomics: A Proof-of-Principle Study

**DOI:** 10.3390/metabo10090343

**Published:** 2020-08-25

**Authors:** Fang Yuan, Seongho Kim, Xinmin Yin, Xiang Zhang, Ikuko Kato

**Affiliations:** 1 Department of Chemistry, University of Louisville, Louisville, KY 40292, USA; fang.yuan@louisville.edu (F.Y.); xinmin.yin@louisville.edu (X.Y.); xiang.zhang@louisville.edu (X.Z.); 2 Department of Oncology, Wayne State University School of Medicine, Detroit, MI 48201, USA; kimse@karmanos.org; 3 Biostatistics Core, Karmanos Cancer Institute, Wayne State University, Detroit, MI 48201, USA; 4 Department of Pathology, Wayne State University School of Medicine, Detroit, MI 48201, USA

**Keywords:** colorectal cancer, untargeted metabolomics, 2DLC-MS, GC × GC-MS, case-control study

## Abstract

Untargeted metabolomics is expected to lead to a better mechanistic understanding of diseases and thus applications of precision medicine and personalized intervention. To further increase metabolite coverage and achieve high accuracy of metabolite quantification, the present proof-of-principle study was to explore the applicability of integration of two-dimensional gas and liquid chromatography-mass spectrometry (GC × GC-MS and 2DLC-MS) platforms to characterizing circulating polar metabolome extracted from plasma collected from 29 individuals with colorectal cancer in comparison with 29 who remained cancer-free. After adjustment of multiple comparisons, 20 metabolites were found to be up-regulated and 8 metabolites were found to be down-regulated, which pointed to the dysregulation in energy metabolism and protein synthesis. While integrating the GC × GC-MS and 2DLC-MS data can dramatically increase the metabolite coverage, this study had a limitation in analyzing the non-polar metabolites. Given the small sample size, these results need to be validated with a larger sample size and with samples collected prior to diagnostic and treatment. Nevertheless, this proof-of-principle study demonstrates the potential applicability of integration of these advanced analytical platforms to improve discrimination between colorectal cancer cases and controls based on metabolite profiles in future studies.

## 1. Introduction

Untargeted metabolomics has been increasingly employed in the past decade for biomarker discoveries for various diseases/conditions as well as for metabolome-wide association studies [1,2]. This is expected to lead to a mechanistic understanding of diseases and thus applications of precision medicine and personalized intervention. Currently, multiple analytical techniques are available for untargeted metabolomics, and they are broadly grouped into gas chromatography (GC) or liquid chromatography (LC) mass spectrometry (MS)-based and nuclear magnetic resonance spectroscopy (NMR)-based studies [1,3,4]. Comparing with NMR, GC-MS and LC-MS are more sensitive and provide much-increased metabolite coverage. However, each of these two methods still has limited resolving power for analysis of complex samples such as metabolites in a biological sample, and therefore none of them is able to analyze all metabolites. Furthermore, it has been reported that metabolites detected by LC-MS and GC-MS are different and that the metabolite overlap and correlations between platforms are modest [5,6,7].

The majority of the untargeted metabolomics studies, thus far, were performed using GC-MS and/or LC-MS, where one-dimensional GC or LC was coupled with MS [8,9]. With the vast complexity and diverse chemical characteristics of metabolites as well as large concentration difference between metabolites in the metabolome, two-dimensional LC or GC coupled with MS have been developed and respectively used in untargeted metabolomics to achieve higher metabolite coverage [9,10]. To further increase metabolite coverage and achieve high accuracy of metabolite quantification, we have developed a method by analyzing the same metabolite sample on comprehensive two-dimensional gas chromatography-mass spectrometry (GC × GC-MS) and two-dimensional liquid chromatography-mass spectrometry (2DLC-MS). We then integrate their data for metabolite identification and quantification [7,11,12]. Such analysis does not only dramatically increase the metabolite coverage but also provide accurate metabolite quantification and reliable metabolic pathway assignment. These advanced techniques should be particularly useful in dealing with complex biological samples [13], such as plasma/serum, which contains not only human endogenous metabolites but also environmental metabolites, plant metabolites, pharmaceutical metabolites, and microbial metabolites in a wide range of concentrations [1]. Yet, the introduction of these advanced two-dimensional approaches to clinical samples has not progressed as rapidly as expected.

Altered energy metabolism, exampled by aerobic glycolysis, has been recognized recently as an emerging hallmark of cancer [14]. Furthermore, metabolic syndrome, consisting of a cluster of abnormal lipid, glucose, and energy metabolisms, predisposes individuals to various cancers [15,16,17]. Colorectal cancer (CRC) is the third most commonly diagnosed cancer in both genders and the second leading cause of cancer deaths in the USA [18]. Although metabolic syndrome is an established risk factor for CRC [15,16,17], there have been few tumor markers for CRC with good diagnostic, predictive, or prognostic values. Carcinoembryonic antigen (CEA) has been routinely used to detect CRC recurrence after surgery but has insufficient sensitivity to be used alone [19]. Thus, new sets of biomarkers would greatly facilitate CRC prevention and patient care. The objectives of the present proof-of-principal study was to explore the applicability of integration of GC × GC-MS and 2DLC-MS platforms to characterizing circulating metabolome in individuals who suffered CRC in comparison with those who remained cancer-free, both limited to non-smokers to minimize influences of tobacco metabolites.

## 2. Results

Our two-dimensional gas chromatography was configured in comprehensive mode, i.e., multiple fractions are collected from the first-dimension column with a modulation period of 2.00 s and each fraction was then subjected to the second-dimension column for further separation. Our 2DLC was configured in parallel mode [20]. Specifically, a sample was first delivered to two sample loops during sample loading. The two sample aliquots were then simultaneously injected onto a dual column setup, and parallel separations were performed on the HILIC and RPC columns, respectively. The eluates from the two columns were then merged and subjected to a mass spectrometer for further analysis. This strategy is simple yet effective for coupling HILIC and RPC to decrease analysis time and increasing throughput. Furthermore, the parallel 2DLC-MS configuration allows the use of two long columns and gradient time to increase separation power and does not suffer from the peak partition and solvent miscibility and solvent strength issues.

GC × GC-MS detected 52 to 126 annotated peaks (i.e., identified metabolites) from each sample with a median of 110, while 2DLC-MS (+) detected 116 to 145 annotated peaks with a median of 127 and 2DLC-MS (−) detected 91 to 137 annotated peaks with a median of 118. When the numbers of annotated peaks were compared by group, the case group had slightly higher numbers than or similar numbers with the control group, i.e., GC × GC-MS (median 112 vs.107), 2DLC-MS (+) (median 127 vs. 127), and 2DLC-MS (−) (median 118 vs.119), respectively, for the cases and controls. It should be noted that each annotated peak in GC × GC-MS refers to a unique metabolite, while two annotated peaks in 2DLC-MS (+) or 2DLC-MS (−) may represent the same metabolite due to parallel 2DLC configuration.

**Figure 1:**
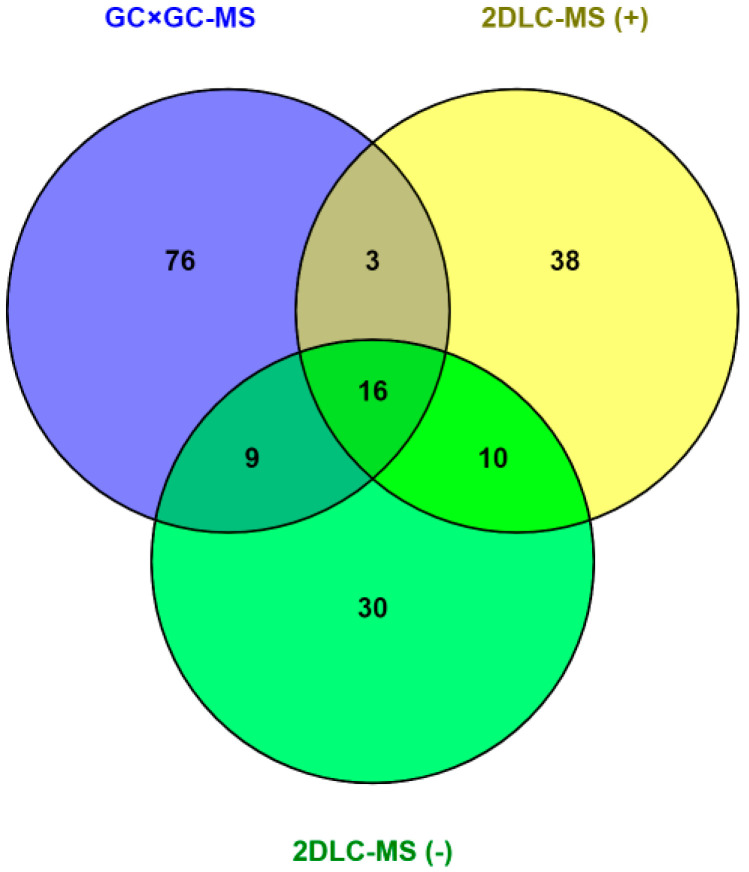
Overlap of metabolites identified from 3 analytical platforms.

**Figure 2:**
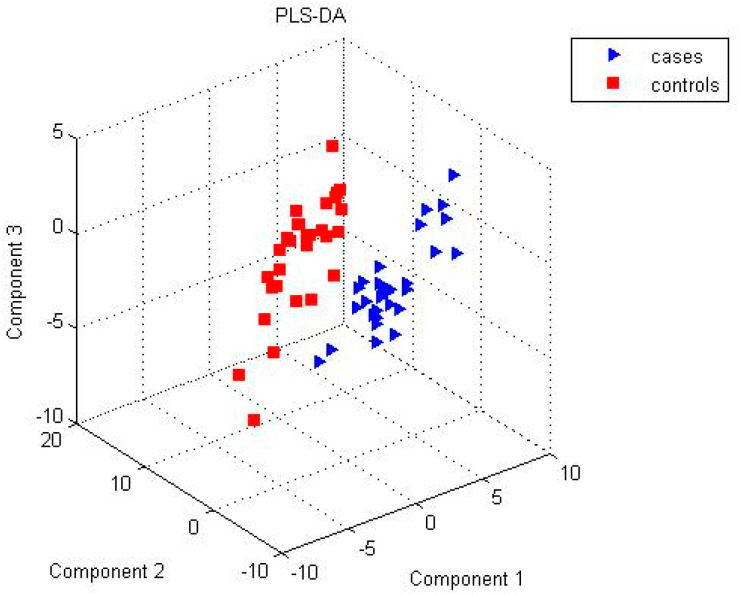
Partial least squares-discriminant analysis (PLS-DA) results of data from 3 platforms.

To study the metabolite identification and abundance changes between groups, we filtered out annotated peaks in the alignment table that presented in < 50% of the samples in each group. After this filtering, the GC × GC-MS dataset had 104 annotated peaks, 2DLC-MS (+) dataset had 112 annotated peaks, and 2DLC-MS (−) dataset had 106 annotated peaks. Figure 1 shows a Venn diagram among unique metabolites identified from multiple platforms after filtering. A total of 16 metabolites were commonly detected by all three platforms, while 19 metabolites by both GC × GC-MS and 2DLC-MS (+), 25 metabolites by both GC × GC-MS and 2DLC-MS (−), and 26 metabolites by both 2DLC-MS (+) and 2DLC-MS (−). Figure 2 shows the results of PLS-DA analysis using the merged data of the three platforms, which confirm that the samples are discriminated by group.

Table 1 shows the results of statistical significance tests, i.e., metabolites with significant abundance changes between cases and controls. After multiple comparison adjustment, 9 metabolites with significant abundance differences were detected by 2DLC-MS (+), and 10 metabolites detected by 2DLC-MS (−) and 9 metabolites detected by GC × GC-MS, with one overlapped metabolite malate between 2DLC-MS (−) and GC × GC-MS. Among these 28 metabolites with different relative abundances, 20 were up-regulated in the cases while 8 were down-regulated. Among the 20 up-regulated metabolites, more than 50% of fold changes were detected for 6 metabolites, including 3-hydroxybutyric acid (2.12), glutamic acid (2.00), beta-alanine (1.76), tricine (1.60), pyroglutamic acid (1.55), malate (1.51), and citric acid (1.51). Among the 8 down-regulated metabolites, the top 5 abundance decline in the cases was observed for propylene glycol (0.50), alanine (0.52), testosterone sulfate (0.54), pinolenic acid (0.56), and piperine (0.63).

**Figure 3:**
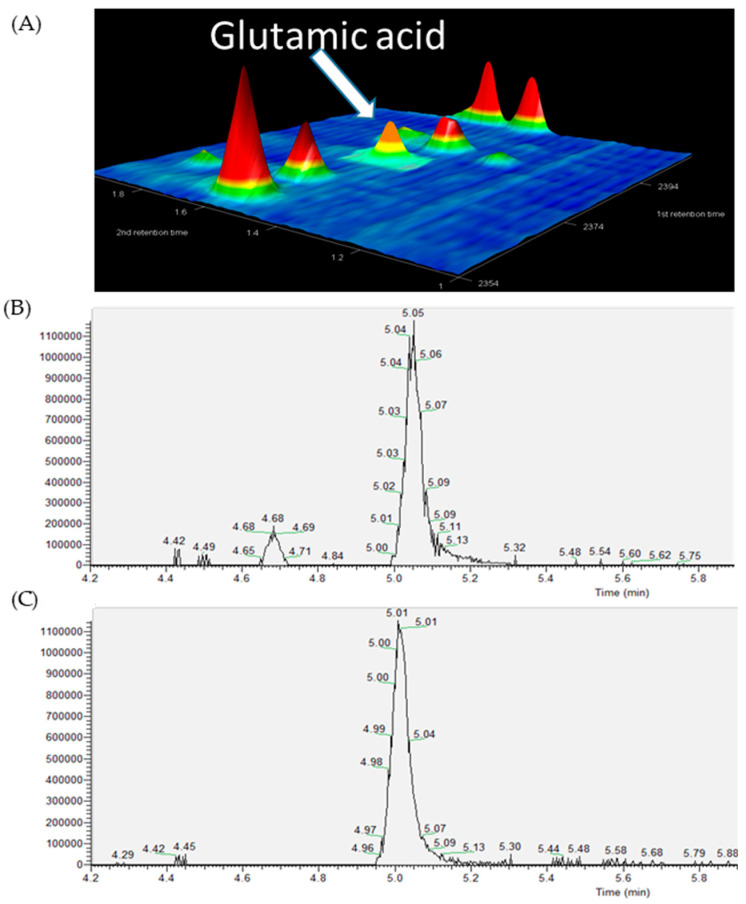
Instrument response of glutamic acid detected by 3 platforms. (**A**) Three-dimensional chromatographic peak of glutamic acid in GC × GC-MS. (**B**) Extracted ion chromatogram of glutamic acid in the same sample detected by two-dimensional gas and liquid chromatography-mass spectrometry (2DLC-MS) (+). (**C**) Extracted ion chromatogram of glutamic acid in the same sample detected by 2DLC-MS (−).

It should be noted that 4 metabolites with significance abundance changes were actually detected in multiple platforms. For example, glutamic acid was detected by all three platforms. While its abundance has significant difference between the cases and the controls in the GC × GC-MS data with *p* = 0.00258 and fold change = 2.00, its abundance changes detected by 2DLC-MS (+) and 2DLC-MS (−) are not significant (*p* = 0.136 and fold change = 1.31 in 2DLC-MS (+); *p* = 0.257 and fold change = 1.19 in 2DLC-MS (−)). However, its abundance change has the same direction, i.e., all up-regulated in the cases. Manual data analysis shows that such discrepancy was mainly caused by the low instrument response (Figure 3), as we reported before [7,11].

**Figure 4:**
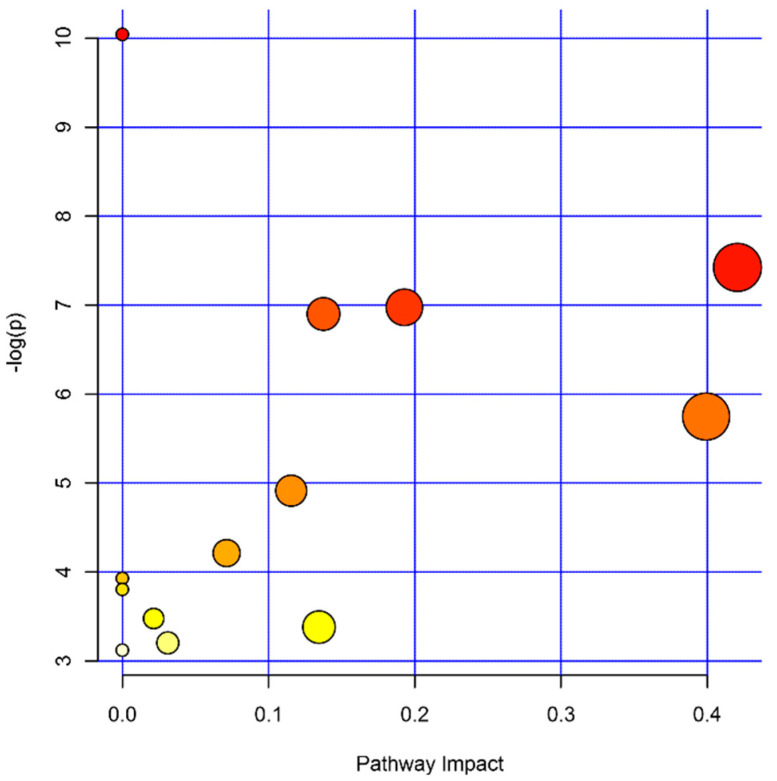
Pathways affected by the metabolic differences between cases and controls. The color and size of circles represent the sizes of the *p*-values (the darker the smaller) and the pathway impact scores (the larger the bigger), respectively.

Combining these three datasets, we further explored the metabolic pathway that was altered by disease status. A total of 13 pathways were detected with a *p* < 0.05. The top five pathways affected by disease history are aminoacyl-tRNA biosynthesis (6 hits), tricarboxylic acid (TCA) cycle (3 hits), glyoxylate and dicarboxylate metabolism (4 hits), beta-alanine metabolism (3 hits), and alanine, aspartate, and glutamate metabolism (4 hits) (Table 2 and Figure 4).

**Figure 5:**
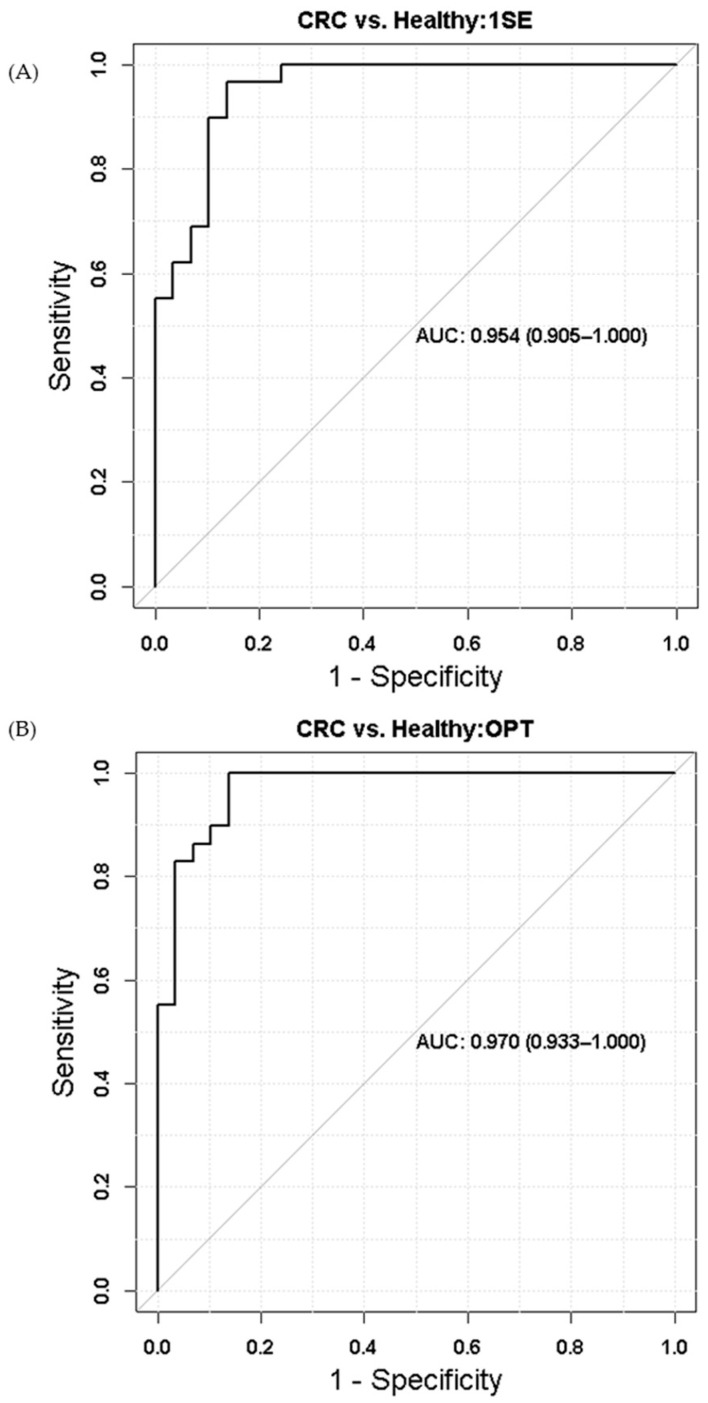
Receiver operating characteristic (ROC) results of (**A**) one standard error (1SE) tuned and (**B**) minimum (OPT) tuned.

Figure 5 depicts the results of validating identified 28 potential metabolite biomarkers (Table 1) using the receiver operating characteristic (ROC) analysis. The metabolites selected for ROC analysis were 4-methyl-2-oxovaleric acid, aspartic acid, beta-alanine, citric acid, glycine, lysophosphatidic acid, neopentyl glycol, oleamide, propylene glycol, testosterone sulfate, and uridine. While 1SE and OPT were respectively used to select an optimal λ in LASSO logistic regression for the development of a prediction model to distinguish CRC cases from control groups, both tuning methods picked up the same set of potential biomarkers as the final list of markers. The area under the curves (AUC) of ROC is 0.954 for 1SE turned (Figure 5A) and 0.970 for OPT tuned LASSO logistic regression (Figure 5B).

## 3. Discussion

Following our previous study focused on volatile organic compounds (VOC) [21], the primary aim of this study was to apply multiple two-dimensional separation techniques with mass spectrometry for metabolite biomarker discovery from complex biological samples by untargeted metabolomics. In the past several years, a number of global metabolomic studies on CRC have been published. These studies utilized diverse biological specimens, including blood (plasma/serum) as the most common type [22,23,24,25,26,27,28,29,30,31,32,33,34,35,36,37,38,39], followed by tissue [27,39,40,41,42], feces [39,40,43], and urine [44,45], while others aimed specifically to detect volatile metabolites in breath and in other types of biospecimens [21,46]. To our knowledge, only two of these were prospective studies where biospecimens were collected before cancer development [26,34], aiming to identify etiological pathways. The rest and ours were based on samples collected after the cancer diagnosis, which was more focused on biomarker discovery. A minority of these studies employed more than one untargeted metabolomic platforms [24,26,31,32,34,43,45], but none of these incorporated multiple two-dimensional separations.

Because metabolic profiles are highly dependent on the choice of the matrix [27], the comparison across different types of biospecimens is not informative. Blood is the most widely used clinical specimens. Hashim et al. [23] recently reviewed the results of blood-based global metabolomic studies for CRC. The authors identified nine studies and found that the number of annotated metabolites in each study vastly varied from 14 [25] to 447 [34], not necessarily in a platform-dependent manner. Each study applied heterogeneous criteria for differential abundance between cases and controls, yielding the number of differential metabolites ranging from 5 [30] to 72 [31]. More recently published studies identified nearly or over 600 named metabolites [26,28], while another recent paper reported a vast number of differentially present metabolites, i.e., 693, in plasma of CRC cases compared with polyp patients [27]. It should be noted that the number of annotated metabolites is affected by many factors, including sample treatments, bioanalytical platforms used for analysis, and the methods of data analysis. Earlier studies with a larger number of annotated metabolites generally used an additional extraction and/or separation technique specifically for lipid metabolites, which are known to be less susceptible to degradation over long-term storage [47].

What was apparent in the aforementioned review and other recent papers on circulating CRC-associated metabolites is that very few metabolites have been detected consistently across the studies. Even if a metabolite was reported in more than one study, often the direction association was different [23]. For instance, there were no lipid metabolites commonly detected in the same direction in recent studies [26,28,29]. The most consistently observed in the same direction as well as by more than one platform were pyruvic acid (up-regulated) and tryptophan (down-regulated) [23]. However, in our study population, neither pyruvic acid nor tryptophan showed significant abundance difference between the cases and the controls, being the fold changes of these metabolites close to unity (0.94–0.99, data not shown).

On the other hand, some of the metabolites differentially present by CRC status in our study were noted by others. Specifically, increased relative abundances of glycine and glutamic acid have been reported in studies [25,30,33,35,40,44]. As in our study, three of these studies [35,40,41] detected increased abundances of both metabolites simultaneously, including two tissue-based studies [40,41] analyzing metabolites directly from CRC but using different analytical platforms (capillary electrophoresis (CE)-TOFMS, NMR, and GC-MS). This indicates that the source of these metabolites is more likely to be cancer itself, rather systemic reactions to the disease. The reported fold changes, if any, have been rather modest (<2.0), consistent with our results. In addition, an increased relative abundance of malate was reported by three serum-based studies with a similar fold change to ours (×1.3) [21,32,36]. Furthermore, increased abundances of uridine and isocitrate were respectively reported in two studies [43,48], and four other studies found a decreased relative abundance of leucine [25,29,30,36].

Based on metabolic pathway analysis, we found five biological pathways were affected by disease history. Among these, alanine, aspartate, glutamate, and β-alanine metabolism pathways are also reported to be dysregulated in both CRC tissue and blood by others [25,27,41,43]. These two metabolic pathways and the TCA cycle are known to be central to fulfill increased energy consumption and increased anabolic activities to sustain cellular growth and proliferation [49]. Glucose and glutamine are the main sources used to maintain active essential metabolic pathways, such as glycolysis and anaplerotic flux of the tricarboxylic acid (TCA) cycle. Dysregulation of the TCA cycle and glutamine addiction are characteristic features of glutamine metabolism in cancers [48]. Glutamate is formed through hydrolysis of glutamine, and it can be further converted to α-ketoglutarate and other TCA intermediates [40,49].

Up-regulation of β-alanine metabolism pathway has been noted in both CRC tissue and plasma by Wang et al. [27]. Besides CRC, increased β-alanine activity was positively associated with an aggressive type of estrogen receptor-negative breast cancer in both tumor tissues and established cell lines [50,51]. β-alanine is crucial for acetyl-CoA synthesis that is vital in TCA cycle and biomass production such as the synthesis of fatty acids, cholesterols, and acetylcholines, fostering cellular metabolism favorable for tumor cell growth [52]. Epithelial-mesenchymal transition induces cancer stem cell characteristics and promotes tumor invasiveness, which was associated with the metabolic reprogramming, including beta-alanine upregulation, and increased β-alanine levels in breast cancer tissue were predictive of overall survival [53]. It is also worth noting that dysregulation of β-alanine metabolism is a common feature of obesity, a known risk factor for CRC [54].

Glyoxylate and dicarboxylate metabolic pathway comprises an important subset of reactions involved in biosynthesis of carbohydrates from fatty acids or two-carbon precursors, which enter the system as acetyl-CoA and thus is closely related to several pathways discussed above [55]. Despite the lack of reports from CRC metabolic steadies, its dysregulation has been described in tumor tissue, peripheral blood, and urine from a variety of cancer-bearing humans and animals including gastric cancer, hepatocellular carcinoma, and myeloid leukemia [55,56,57]. This was confirmed in pathway analysis based on gene expression of aggressive breast cancer [58]. Thus, dysregulation of this pathway may represent a common metabolic profile of advanced cancer.

Our data also pointed to the dysregulation of the aminoacyl-tRNA biosynthesis pathway CRC patients. Aminoacyl-tRNA synthetases (ARSs) carry out the first step of protein translation by catalyzing the litigation of amino acids to their cognate tRNAs. Aberrant expression, mislocalization, and variant formation of ARSs have been recently observed in various cancer cells [59,60]. However, it has not been clear whether increased expression of ARSs in cancer tissue/cells results from the increased demand for protein synthesis in cancer cells or whether ARSs are drivers of cell transformation and growth. Overexpression of specific types of ARSs has also been noted. Particularly, an increase in the catalytic activity of methionyl-tRNA synthetase (MRS) that is required for translation initiation was reported in human colon cancer [61] and experimental overexpression of methionyl-tRNA in breast epithelial cell lines resulted in increased metabolic activity and cell proliferation [62]. Leucyl-tRNA synthetase overexpression has been linked to activation of mammalian target of rapamycin complex 1 (mTORC1)-signaling pathway that controls critical cellular processes, including cell growth, metabolism, and autophagy, and thus contributes to carcinogenesis [63]. In addition, leucyl-tRNA-derived small RNA was found essential for cell viability and tumor growth mediating ribosome biogenesis [64]. Furthermore, it has become clear that ARSs possess versatile non-canonical functions, interacting with diverse regulatory networks and fine-tuning post-transcriptional gene expression, which is relevant to various pathological conditions, including cancer [60,61,62,63].

While metabolomic profiling has been informative in identifying new therapeutic targets, the clinical utility of selected metabolomic markers has been tested in early detection of colorectal cancer [33,36]. Although most single metabolites showed poor to moderate values of area under the curves (AUC), high AUC values (>0.90) were achieved using a panel of selected metabolites in our and other studies. However, actual clinical translational values of this approach are still questionable. Metabolite abundance obtained from an untargeted metabolomic study is generally normalized relative values. To be used as a clinical test, cut-offs based on absolute concentrations are necessary. More importantly, little has been known about changes in these endogenous metabolites by physiological or pathological conditions other than colorectal cancer. It is possible that identified metabolites are merely markers for underlying obesity, inflammation, or aging [65,66,67]. Furthermore, since circulating levels of the metabolites are expected to be much lower than those in cancer tissue are and since these endogenous metabolites are not tissue-specific, high sensitivity will be required to detect changes in peripheral blood arising from the small early stage of cancer.

The strengths of current study include the controls derived from the same general population that gave rise to the cases, availability of the information of detailed personal characteristics, and integration of multiple advanced separation techniques in global metabolomics. We acknowledge limitations in our study. Being an early stage of the study, the sample size was small to obtain estimates with high precision. Besides, as biological samples used in this study were collected after cancer treatment/resection from the cases, the direct effect of the presence of the primary tumor was deemed to have faded, although underlying metabolic deviations that fostered cancer development were expected to remain. In fact, resection of CRC does not normalize metabolic shifts seen in breath VOCs [68] or in blood total metabolites [29]. Although the storage at −80 °C is considered appropriate for most metabolomic studies, metabolite degradation over the long-term storage is certainly possible [33]. However, it is reasonable to assume that the degradation would occur similarly over time between cases and controls. Yet, we acknowledge that the discriminating power may decline with longer storage due to the degradation of low abundance metabolites. Finally, the integration of lipid profiling, which was missing in this study, would provide a more comprehensive view of cancer-causing or cancer-induced metabolomic abnormalities

## 4. Materials and Methods

### 4.1. Materials

Sodium phosphate dibasic (Na_2_HPO_4_, purity 100.2%, Cat. No. S374-500), sodium phosphate monobasic (NaH_2_PO_4_, purity 99%, Cat. No. 389872500), and sodium hydroxide (NaOH, Cat. No. S399-500) were purchased from Fisher Scientific Inc. (Pittsburgh, PA, USA). Dichloromethane (CH_2_Cl_2_, purity ≥ 99.8%, Cat. No. 34856-1L), methoxyamine hydrochloride (purity ≥ 98%, Cat. No. 226904-5G), and a mixture containing C_7_–C_26_ *n*-alkanes (purity 95–99%, Cat. No. 49451U) were purchased from Sigma-Aldrich Corp. (St. Louis, MO, USA). *N*-methyl-*N*-[*tert*-butyldimethylsilyl trifluoroacetamide (MTBSTFA) with 1% tert-butyldimethylchlorosilane (TBDMCS) was purchased from Restek Corp. (Bellefonte, PA, USA). dH_2_O was prepared from a Millipore synergy system (Burlington, MA, USA).

### 4.2. Study Samples

The samples used in this study were plasma samples collected for a population-based CRC case-control study. It was conducted in Metropolitan Detroit during the period of 2003–2006 and designed to enroll frequency-matched cases and controls according to their gender, age, and county. The study was approved by the Institutional Review Board of Wayne State University. Details concerning the parent study were described elsewhere [69]. Blood was collected in ethylenediaminetetraacetic acid tubes at subjects’ home or other preferred locations and delivered to a processing laboratory with cooling gels where plasma samples were separated, aliquoted, and stored in −80 °C freezers. Dietary and other personal characteristics were obtained by the use of structured questionnaires. A total of 1421 subjects who were not smoking for at least preceding two years, either Caucasian or African American, donated blood samples in the parent study.

For this study, 30 cases and 30 controls were randomly selected from the original pool of the parent study, stratified by race and gender (50% white/black; 50% male/female), as well as no cancer targeted therapies (surgery, radiation, or chemotherapy). Because the freeze-thaw cycle affects the stability of metabolites, we used samples not previously thawed for this study. The mean age of both cases and control groups was equally 64 years old. Among selected, two misretrieved samples (one case and one control) were removed from the analysis, leaving 29 cases and 29 controls.

### 4.3. Sample Preparation

To extract polar metabolites from a plasma sample, 400 µL methanol was mixed with 100 µL plasma. After 2 min of vigorously vortex-mixing, the mixture was centrifuged at 4 °C, 14,000 rpm for 20 min. The supernatant was aliquoted into two 200 µL aliquots, and both aliquots were dried by speed vacuum at 4 °C to remove methanol followed by freeze-drying to remove water. Dried aliquots were stored at −80 °C until use. One aliquot was redissolved in 150 µL 20% acetonitrile for 2DLC-MS analysis, while the other aliquot was used for GC × GC-MS analysis after being derivatized by MTBSTFA.

For derivatization, the dried aliquot was dissolved in 30 μL methoxyamine hydrochloride (MH) solution (20 mg/mL in pyridine) and vigorously vortex-mixed for 2 min. Methoxylation was carried out at 60 °C for 1 h. Then, 30 μL MTBSTFA with 1% TBDMCS was added to the sample. Derivatization was carried out at 60 °C for 1 h. After centrifugation at 20 °C, 3000 rpm for 10 min, the supernatant was transferred into a GC vial. Methoxylation and derivatization were conducted right before GC × GC-MS analysis. For quality control, a pooled sample was prepared in the same way by mixing a small amount of each sample. The C7–C26 *n*-alkane mixture was dissolved in dichloromethane at a concentration of 20 µg/mL per compound and analyzed to convert retention time into retention index.

### 4.4. GC × GC-MS Analysis

GC × GC-MS analysis was performed on a LECO Pegasus 4D GC × GC-Time of Flight (TOF) system (LECO Corporation, Saint Joseph, MI, USA) consisting of a Gerstel MPS2 auto-sampler (GERSTEL Inc., Linthicum, MD, USA), an Agilent 6890 gas chromatography (Agilent Technologies, Santa Clara, CA, USA), a LECO two-stage cryogenic modulator, and a LECO Pegasus time-of-flight mass spectrometer with an electron ionization (EI) ion source. The first-dimension column was a J&W DB-5ms Ultra Inert column (60 m × 0.25 mm × 0.25 μm) and the second-dimension column was a J&W DB-17 column (1 m × 0.1 mm × 0.1 μm). Both columns were purchased from Agilent Technologies. The sample injection volume was 1 µL, with a split ratio of 10. The carrier gas was helium (99.999% purity) at 2.0 mL/min corrected constant flow via pressure ramps. The temperature of the inlet and the transfer line were all set to 280 °C. The primary oven temperature ramp for the first-dimension column was 60 °C for 0.5 min, then 5 °C/min to 270 °C, and hold for 15 min at 270 °C. The secondary oven temperature ramp for the second-dimension column was 10 °C warmer with the same temperature gradient as the primary oven. The modulator temperature was 15 °C warmer than the secondary oven, and modulation period was set to 2.00 s. The mass acquisition range was 29–800 Da at an acquisition rate of 200 spectra/s. The ion source temperature was 230 °C. The detector voltage was 1521 V with an electron energy of 70 eV. The acceleration voltage was turned on after a solvent delay of 645 s.

### 4.5. 2DLC-MS Analysis

2DLC-MS analysis was performed on a DIONEX UltiMate 3000 UHPLC system coupled with a Q Exactive HF Hybrid Quadrupole-Orbitrap mass spectrometer (Thermo Fisher Scientific, Inc., Langenselbold, Germany). A reversed-phase chromatography (RPC) column and a hydrophilic interaction chromatography (HILIC) column was configured in parallel mode in the LC system [21]. The RPC column was ACQUITY UPLC HSS T3 (150 × 2.1 mm, 1.8 μm, Waters Corporation, Milford, MA, USA) and the HILIC column was SeQuant ZIC-cHILIC (150 × 2.1 mm, 3 μm, Merck KGaA, Darmstadt, Germany) equipped with a guard column. The LC conditions were set as follows: mobile phase A was water with 0.1% formic acid for PRC and 10 mM ammonium acetate (pH adjusted to 3.25 with acetate) for HILIC. The mobile phase B was acetonitrile with 0.1% formic acid for both RPC and HILIC. The RPC gradient was 0 min, 5% B, hold for 5.0 min; 5.0 min to 6.1 min, 5% B to 15% B; 6.1 min to 10.0 min, 15% B to 60% B, hold for 2.0 min; 12.0 min to 14.0 min, 60% B to 100% B, hold for 13.0 min; 27.0 min to 27.1 min, 100% B to 5% B, hold for 12.9 min. The HILIC gradient was 0 to 5.0 min, 95% B to 35% B, hold for 1.0 min; 6.0 min to 6.1 min, 35% B to 5% B, hold for 16.9 min; 23.0 min to 23.1 min, 5% B to 95% B, hold for 16.9 min. The flow rate was 0.4 mL/min for RPC or 0.3 mL/min for HILIC. The column temperature was 40 °C for both RPC and HILIC.

Under the same LC conditions, all samples were firstly analyzed in full MS scan mode in a random order to obtain MS data for metabolite quantification. Samples from the same group were then combined as pool samples and analyzed in full MS/dd-MS^2^ scan mode under collision energy of 20, 40, and 60 eV, respectively. Both MS and MS/MS data were collected in negative and positive ionization modes using parameters recommended by the vendor (Supplemental Table S1).

### 4.6. Data Analysis

GC × GC-MS data were first analyzed by LECO’s instrument control software ChromaTOF (version 4.51.6.0) using parameters recommended by the vendor (Supplemental Table S2). Metabolites were initially assigned by mass spectral matching using the NIST/EPA/NIH mass spectral library (version 2.2) as reference. Any metabolite assignment with a similarity score below 500 (out of 1000) was discarded. The analysis results of each sample were exported as a peak list containing the information of top 10 metabolite assignments for each chromatographic peak. All peak lists were further analyzed by MetPP software for retention index (RI) matching, peak merging, and cross-sample peak list alignment [64,70]. The initially assigned metabolites were further filtered by RI matching with a threshold of *p* ≤ 0.001 (equivalent to an absolute RI window of ±59 RI units). To further reduce false positives, different chromatographic peaks with the same metabolite identification were removed except peaks that were verified by experiment information of authentic standards recorded in our in-house database.

The 2DLC-MS data were first converted into mzXML format by MSConvert software (Proteowizard, Palo Alto, CA, USA). The mzXML files were then subjected to MetSign software for spectrum deconvolution and cross-sample peak list alignment [71,72,73]. Metabolite identification was achieved using the 2DLC-MS/MS data of the pooled samples, as described in our previous work [7,11,12]. Briefly, MS/MS spectra of pool samples were first matched with the information of authentic standards of metabolites recorded in our in-house database, including parent ion *m/z*, retention time, and MS/MS spectrum similarity with thresholds of parent ion *m/z* variation < 5 ppm, retention time variation < 0.15 min, and MS/MS spectral similarity > 0.4. The experimental data of MS/MS spectra that do not have a match in the in-house database were further subjected to Compound Discoverer v2.0 (Thermo Fisher Scientific Inc., Langenselbold, Germany), where metabolite identification was achieved by parent ion matching with *m*/*z* variation < 5 ppm and MS/MS spectrum matching with a similarity threshold > 40 (out of 100).

After metabolite identification, data collected from three platforms, GC × GC-MS, 2DLC-MS (+), and 2DLC-MS (−) were merged and normalized by contrast-based normalization using Metsign software [71,72,73]. Partial least squares-discriminant analysis (PLS-DA) was performed to study the metabolic profile difference among groups. A pairwise two-tail *t*-test was carried out to determine whether a metabolite has a significant difference in abundance between two groups. Grubbs’ test was employed for outlier detection before the *t*-test, and the outliers were excluded for the *t*-test. After the *t*-test, the *p*-values were adjusted for multiple comparisons using permutation-based adjustments with 1000 bootstraps. A metabolite was considered to be significantly different in abundance between two groups if it was detected in more than 50% of samples in each group and had an adjusted *p* < 0.05.

We further validated identified potential metabolomic markers using the receiver operating characteristic (ROC) analysis. We used a penalized logistic regression for the development of a prediction model to distinguish CRC cases from control groups using the least absolute shrinkage and selection operator (LASSO) approach. The LASSO logistic regression has a tuning parameter. The lambda (λ), a tuning parameter of the LASSO logistic regression, was tuned using leave-one-out cross-validation. When selecting an optimal λ, we used two methods, one standard error (1SE) and minimum (OPT), based on the deviance (i.e., mean squared error). The method OPT selects the value of lambda with the smallest deviance, while the method 1SE chooses the value of lambda that minimizes the deviance plus one standard error.

Pathway analysis was performed using Metaboanalyst (version 4.0) [74]. Names of metabolites identified with significant abundance differences were uploaded and analyzed by the program. The data were matched against the KEGG pathway library for *homo sapiens*. The “Hypergeometric Test” and “Relative-betweenness Centrality” algorithms were, respectively, used for over-representation and pathway topology analysis.

## 5. Conclusions

This proof-of-principle study tested the integration of two two-dimensional GC- and LC-MS for metabolite biomarker discovery for colorectal cancer using archived human plasma specimens. After adjustment of multiple comparisons, 20 metabolites and 8 metabolites were found to be up-regulated and down-regulated, respectively. The metabolites with 50% or higher increase in cancer cases than controls included 3-hydroxybutyric acid, glutamic acid, beta-alanine, tricine, pyroglutamic acid, malate, and citric acid. These metabolites with significant abundance differences also pointed to the dysregulation in energy metabolism and protein synthesis. While integrating the GC × GC-MS and 2DLC-MS data could dramatically increase the metabolite coverage, we only analyzed the polar metabolites in this study. Given this limitation, caution should be excised not to over-interpret our results. Furthermore, due to the small sample size, these results need to be validated with a large sample size and with samples collected prior to diagnostic and treatment. Nevertheless, this proof-of-principle study demonstrates the potential applicability of integration of these advanced analytical platforms to improve discrimination between colorectal cancer cases and controls based on metabolite profiles in future studies.

## Figures and Tables

**Table 1:** Metabolites with significant abundance change between cases and controls.

Name	*p*-Value	Fold Change (Cases/Controls)	Platform	%FO *	
				Controls *n* = 29	Cases *n* = 29
3-Hydroxybutyric acid	9.98 × 10^−3^	2.12	2DLC-MS (−)	96.55	96.55
4-Methyl-2-oxovaleric acid	4.62 × 10^−3^	0.71	2DLC-MS (−)	100.00	100.00
Adonitol	1.07 × 10^−2^	1.27	2DLC-MS (−)	62.07	62.07
Alanine	4.98 × 10^−3^	0.52	2DLC-MS (−)	100.00	100.00
Arginine	3.83 × 10^−2^	0.78	2DLC-MS (+)	82.76	82.76
Aspartic Acid	5.09 × 10^−3^	0.74	2DLC-MS (+)	79.31	79.31
beta-Alanine	4.15 × 10^−2^	1.76	GC × GC-MS	100.00	100.00
Choline	8.56 × 10^−3^	1.19	2DLC-MS (+)	68.97	72.41
Citric acid	2.13 × 10^−2^	1.51	2DLC-MS (−)	100.00	100.00
Ethanolamine	3.17 × 10^−2^	1.31	2DLC-MS (+)	100.00	100.00
Glutamic acid	2.58 × 10^−3^	2.00	GC × GC-MS	68.97	72.41
Glutaric acid	4.86 × 10^−2^	1.16	2DLC-MS (−)	96.55	96.55
Glycine	1.44 × 10^−3^	1.34	2DLC-MS (+)	93.10	89.66
Hydracrylic acid	4.32 × 10^−2^	1.28	GC × GC-MS	100.00	100.00
Lactic acid	3.11 × 10^−2^	1.13	GC × GC-MS	100.00	100.00
Lysine	1.86 × 10^−2^	1.30	GC × GC-MS	79.31	82.76
Lysophosphatidic acid	4.57 × 10^−2^	1.23	2DLC-MS (+)	100.00	100.00
Malate	2.09 × 10^−2^	1.37	2DLC-MS (−)	100.00	100.00
Malate	4.91 × 10^−2^	1.51	GC × GC-MS	72.41	72.41
Neopentyl glycol	3.24 × 10^−2^	1.16	GC × GC-MS	100.00	100.00
Oleamide	3.40 × 10^−2^	1.11	2DLC-MS (+)	72.41	72.41
Pinolenic Acid	2.26 × 10^−2^	0.56	2DLC-MS (+)	72.41	75.86
Piperine	4.29 × 10^−2^	0.63	2DLC-MS (+)	100.00	100.00
Propylene glycol	4.03 × 10^−3^	0.50	GC × GC-MS	96.55	100.00
Pyroglutamic acid	1.21 × 10^−2^	1.55	2DLC-MS (−)	72.41	72.41
Testosterone sulfate	2.83 × 10^−2^	0.54	2DLC-MS (−)	100.00	100.00
Tricine	1.81 × 10^−2^	1.60	GC × GC-MS	86.21	82.76
Uridine	2.48 × 10^−2^	1.25	2DLC-MS (−)	96.55	96.55

* FO: Percentage frequency of occurrence.

**Table 2:** Pathways affected by the metabolic differences between cases and controls.

Name	Total	Hits	*p*-Value	Impact
Aminoacyl-tRNA biosynthesis	48	6	4.35 × 10^−5^	0
Alanine, aspartate, and glutamate metabolism	28	4	5.97 × 10^−4^	0.42068
Arginine biosynthesis	14	3	9.35 × 10^−4^	0.19289
Glyoxylate and dicarboxylate metabolism	32	4	1.01× 10^−3^	0.13757
beta-Alanine metabolism	21	3	3.19 × 10^−3^	0.39925
Glutathione metabolism	28	3	7.34 × 10^−3^	0.11548
Glycerophospholipid metabolism	36	3	1.48 × 10^−2^	0.07130
Butanoate metabolism	15	2	1.97 × 10^−2^	0
Histidine metabolism	16	2	2.23 × 10^−2^	0
Pantothenate and CoA biosynthesis	19	2	3.09 × 10^−2^	0.02143
Citrate cycle (TCA cycle)	20	2	3.40 × 10^−2^	0.13450
Pyruvate metabolism	22	2	4.06 × 10^−2^	0.03110
Propanoate metabolism	23	2	4.41 × 10^−2^	0.00000

## Supplementary Materials

The following are available online at https://www.mdpi.com/2218-1989/10/9/343/s1, Table S1: Mass spectrometer parameters for 2DLC-MS analysis. Table S2: ChromaTOF software parameters for GC × GC-MS analysis.
