# Supplementary material for: Integrating Two-Dimensional Gas and Liquid Chromatography-Mass Spectrometry for Untargeted Colorectal Cancer Metabolomics: A Proof-of-Principle Study
Source: Metabolites. 2020 Aug 25;10(9):343. doi: 10.3390/metabo10090343 (PMC7569982; doi:10.3390/metabo10090343)
Supplement: Supplementary file 1 [file metabolites-10-00343-s001.pdf]

Integrating two-dimensional gas and liquid chromatography-mass spectrometry for untargeted colorectal cancer metabolomics: a proof-of-principle study

Fang Yuan,[1] Seongho Kim,[2,3] Xinmin Yin,[1] Xiang Zhang,[1] and Ikuko Kato[2, 4 *]

***Corresponding Author**

Supplemental Data

Table S1. Mass spectrometer parameters for 2DLC-MS analysis

General and full MS parameters		dd-MS^2 parameters	
Runtime	0 to 20 min	Microscans	2
Polarity	negative/positive	Resolution	15,000
In-source CID	0.0 eV	ACG target	5e4
Microscans	2	Maximum IT	100 ms
Resolution	30,000	Loop count	6
ACG target	1e6	MSX count	1
Maximum IT	50 ms	Isolation window	0.4 m/z
Number of scan ranges	1	Isolation offset	0.0 m/z
Scan ranges	60 to 900 m/z	(N)CE/stepped (N)CE	nce:10/20/40/60
Spectrum data type	Centroid	Spectrum data type	Centroid
		Minimum ACG target	8.00e3
		Exclude isotopes	on
		Dynamic exclusion	1.2 s

Table S2. ChromaTOF software parameters for GC×GC-MS analysis

Parameters	Setting values
Baseline offset	1.0 (just above the noise)
Number of data points averaged for smoothing	Auto
First dimension peak width	12 s
Second dimension peak width	0.1 s
Match required to combine	600
Minimum S/N	10
Mass to use for area/height calculation	Unique mass
Integration approach	Traditional
Minimum/maximum molecular weight allowed	29/800
Mass threshold (relative abundance of base ion)	5
Libraries to use for searching	NIST-14
Minimum similarity match before name is assigned	0
Maximum number of unknown peaks to find	5000
Number of library hits to returned	10
Exported information	Name, CAS, 1st dimension time (s), 2nd dimension time (s), similarity, quant masses, area, S/N
